# Recent advances in pelvic floor repair

**DOI:** 10.12688/f1000research.15046.1

**Published:** 2019-06-04

**Authors:** Emma Mironska, Christopher Chapple, Sheila MacNeil

**Affiliations:** 1 Department of Materials Science and Engineering, Kroto Research Institute, University of Sheffield, Red Hill, Sheffield, S37HQ, UK; 2 Urology Department, Royal Hallamshire Hospital, Glossop Road, Sheffield, S10 2JF, UK; 3 Department of Materials Science and Engineering, Kroto Research Institute, University of Sheffield, Red Hill, Sheffield, S37HQ, UK

**Keywords:** pelvic organ prolapse, stress urinary incontinence, POP, SUI, tissue engineering, mesh, incontinence, prolapse, womens health, urology, gynaecology

## Abstract

Stress urinary incontinence (SUI) and pelvic organ prolapse (POP) are conditions which result in significant physical, mental and social consequences for women worldwide. The high rates of recurrence reported with primary repair for POP led to the use of synthetic mesh to augment repairs in both primary and secondary cases following failed previous POP repair. The widely reported, unacceptably high rates of complications associated with the use of synthetic, transvaginal mesh in pelvic floor repair have severely limited the treatment options that surgeons can offer. This article summarises the recent advances in pelvic floor repair, such as improved quantification and modelling of the biomechanics of the pelvic floor and the developing technology within the field of tissue engineering for treatment of SUI/POP, including biomaterials and cell-based therapies. Finally, we will discuss the issues surrounding the commercial introduction of synthetic mesh for use within the pelvic floor and what lessons can be learned for the future as well as the current guidance surrounding treatment for SUI/POP.

## Introduction

Stress urinary incontinence (SUI) and pelvic organ prolapse (POP) are common conditions affecting women worldwide and are associated with significant morbidity and impact on quality of life. It is estimated that 10% of parous women in the UK will go on to require surgical intervention for pelvic floor dysfunction ^1^.

Historically, the surgical options for SUI and POP relied on directly repairing the patient’s own native tissues by using sutures ^2^ or repositioning strips of the patient’s fascia to provide support ^3^. The Burch colposuspension uses a retropubic approach to resuspend the bladder neck back to the correct anatomical location by using sutures. For POP, surgeons used sutures to strengthen the walls of the vagina or to return the pelvic organs to their natural positions. The options for SUI included the use of an autologous fascial sling, which was popularised by McGuire in the 1970s ^2^. Here, a strip of rectus fascia or fascia lata is used to provide support to the urethra.

The prevalence of SUI and POP typically increases with age, and the peak incidence is in post-menopausal, multiparous women. Several other factors (related to both lifestyle and genetics), including obesity ^3^ and smoking and connective tissue disorders ^4^, may contribute to pelvic floor instability. As such, the recurrence and reoperation rates for prolapse with native tissue repair alone were high (failure rate of 17 to 20% at 10 years ^5^) as the patient’s tissues often are not of sufficient quality or strength to be repaired. To circumvent this issue, mesh began to be used to strengthen the repair. The most common material used in these surgeries has been synthetic, polypropylene (PPL) mesh, which is used to support either the urethra (for SUI) or the pelvic organs (for POP). In the case of SUI, this is via the placement of a mid-urethral sling/tape to provide support to the urethra. For POP repair, the repair technique differs depending on the type of prolapse. Broadly speaking, two surgical approaches can be used: abdominal and transvaginal. Sacrocolpopexy and sacrohysteropexy use the abdominal placement of mesh from the sacrum to the vagina or uterus to restore the organs to their natural positions ^6^. Both operations can be performed through open surgery but are more commonly laparoscopic. Colporrhaphy is an open, transvaginal repair using absorbable sutures to plicate the tissues of the anterior or posterior walls (or both) of the vagina and mesh to reinforce the repair. The goals are to provide extra support to the pelvic floor for the overlying organs and prevent further prolapse of either the bladder (cystocele) or rectum (rectocele) into the vagina ^7^.

The use of mesh in pelvic floor repair was standard practice for many years prior to the release of a public health notification by the US Food and Drug Administration (FDA) in 2008 ^8^. This highlighted that they had received an alarmingly high number of reports (more than 1000) concerning complications associated with transvaginal mesh placement in SUI and POP in the preceding 3 years. Subsequent releases confirmed that these initial reports were not an anomaly and that many patients worldwide were experiencing serious adverse events as a result of mesh implantation ^9^. These complications included recurrence of SUI/POP, pain, infection and mesh erosion which have left many women unable to function in their everyday lives. This has led to huge lawsuits against both manufacturers and individual surgeons; as a result, many device manufacturers have dropped pelvic mesh products entirely. Although it is difficult to quantify the exact total cost of litigation due to out-of-court settlements, Endo International Plc (a global pharmaceutical company that purchased American Medical Systems in 2011 ^10^, thereby inheriting the subsequent litigation associated with their mesh products) has set aside $2.6 billion in reserves to deal with mesh lawsuits ^11^. This is now acknowledged as an important issue in healthcare. Future developments of new products in this market quite correctly will require intense regulatory scrutiny.

## Biomaterials

The synthetic, biological, biodegradable or non-biodegradable options currently in use or under study for use within the pelvic floor are considered below. A number of polymers have been developed and have been used in this context. Unfortunately, to many, the word *polymer* has become synonymous with the word *plastic*. This is an oversimplification of a broad range of materials. A polymer is a substance composed of macromolecules ^12^. Plastics are a group of synthetic polymers, often with other chemicals and colouring agents added. All plastics are polymers certainly, but not all polymers are plastics. It is important to note that many different polymers exist in nature (for example, cellulose and even DNA). Many different polymers have been used to investigate pelvic floor repair. These include but are not limited to polylactic acid (PLA) ^13– 20^, polylactic-co-glycolic acid (PLGA) ^21– 24^, polyamide ^25, 26^, purified collagen gel (PPC) ^27^, polyurethane (PU) ^16, 28^, polyvinylidene fluoride (PVDF) ^15^, polycaprolactone (PCL) ^22, 29^ and PPL ^30, 31^.

PPL is a widely used polymer and is used in products ranging from packaging, clothing, car bumpers to toys as well as a multitude of medical components ^32^. Indeed, owing to its chemical stability and non-biodegradable nature, it is the most common material used in synthetic mesh production. PPL meshes have a monofilament structure, are macroporous and have a large enough pore size to allow host cell infiltration. The rationale behind this is that host cell infiltration allows better tissue integration and therefore improved healing and union. PPL meshes were first used for abdominal wall hernia repair, and similar issues of pain and shrinkage were reported during their introduction. In studies in dogs, Klinge *et al*. ^33^ identified a foreign body reaction and persistent inflammation surrounding the PPL meshes, and there was extensive fibrosis and 30 to 50% shrinkage in the first month after implantation ^34^.

In response to the complications associated with PPL mesh in the pelvic floor, manufacturers have produced both “light weight” versions and other modified PPL meshes to attempt to reduce the excessive fibrosis and inflammation which are likely to contribute to exposure in the vagina or erosion through the pelvic organs. Feola *et al*. ^31^ compared the host response to several commercially available PPL meshes in sheep, including meshes with added collagen coatings. Their study found that the addition of collagen did not improve graft-related complication rates. Hachim *et al*. ^35^ modified commercially available PPL meshes with an interleukin-4 (IL-4)-releasing coating. They found that this coating was able to shift host macrophages towards an M2 macrophage response, thus mitigating the foreign body response usually seen with PPL.

PLA is a commonly used polymer in tissue engineering. PLA medical devices are already used in surgery, particularly within orthopaedics. PLA is produced by the bacterial fermentation of carbohydrates; typically, this is corn starch on an industrial scale ^36^. It is renewable and biodegradable and degrades by hydrolysis into lactic acid, which is readily metabolised within the body. De Tayrac *et al*. ^13^ demonstrated that a PLA mesh retained acceptable strength *in vitro* for 8 months and demonstrated good biocompatibility *in vivo* after 90 days of implantation in an incisional hernia rat model. PLA is extremely biocompatible, but given its biodegradability, there are concerns that PLA may not provide the long-term structural support required for a successful pelvic floor repair.

Electrospinning is a technique used to manufacture polymer scaffolds. It works by using electricity to produce fibres from polymer solutions, weaving them into nanofibrous mats ^37^. PLA can be used as a drug delivery system through the method of emulsion electrospinning. This allows the incorporation of hydrophilic substances into PLA fibres. Mangir *et al*. ^17^ successfully produced PLA scaffolds that released two derivatives of ascorbic acid. Fibroblasts then were seeded onto each scaffold and were found to produce more collagen in the presence of ascorbic acid compared with cells seeded onto control scaffolds. In further work, Mangir *et al*. ^14^ developed PLA scaffolds that released controlled doses of oestradiol. These scaffolds were found to increase the extracellular matrix production of seeded adipose-derived mesenchymal stem cells (ADMSCs) and stimulate angiogenesis. Electrospun PU scaffolds have since been augmented with the addition of oestradiol, which has been shown to remarkably promote angiogenesis and improve the mechanical properties of the scaffold ^28^.

Biological grafts have also been used in the pelvic floor, and several commercial xenografts, including porcine small intestinal mucosa, bovine dermis or pericardium, have been produced. These tissues undergo extreme processing and sterilisation measures to reduce the risk of both rejection and infection but this does result in rapid degradation. The materials are non-porous and do not allow continuous host cell integration, leading to poor tissue union. As such, similar to PPL, they often induce a fibrotic reaction. Systematic reviews have not found an advantage to using xenografts over native tissue repair or light-weight synthetic mesh ^38^. Cadaveric dermal allografts have also been used previously ^39^ but these are costly and entail a small but theoretical risk of blood-borne virus transmission. Seitz *et al*. compared patient outcomes for those undergoing hysteropexy augmented with PPL mesh versus hysteropexy with cadaveric dermal allograft ^39^. This study found that the PPL group had a lower recurrence rate compared with the allograft group (18% recurrence rate with mesh and 29% recurrence rate with allograft), but there was a mesh exposure rate of 5.75% in the former group ^39^.

MatriStem™ is a commercially available extracellular matrix bio-scaffold derived from the porcine urinary bladder and is used topically for wound management ^40^. This was used by Liang *et al*. ^41^ in monkeys to repair transected uterosacral ligaments and paravaginal attachments to the pelvic side wall. Matristem™ was used to repair the transected ligaments through either a transvaginal or transabdominal incision. These animals were then compared to others who had the same operation as the Matristem™ implanted animals (laparotomy +/- vaginal incision) but did not undergo “disruption of level I and II support or the application of the bioscaffold”. The authors found that, in comparison with these ‘sham-operated’ controls, there were new tissue bands that had replaced the implanted material. This does raise the possibility that this material could be used for the regeneration of damaged supportive tissues within the pelvic floor in women with POP but clearly more studies need to be performed for this indication.

The choice of material for use in the pelvic floor is vitally important as it must be able to withstand stress and strain but should also be flexible and have inherent elasticity. This is important to prevent the material from deforming and losing its designed structure and from being so stiff as to erode through a patient’s own tissues or even into the viscera. The pelvic floor is an area of the body that undergoes significant movement and morphological changes throughout a woman’s lifetime and any implanted material would need to be able to cope with the stressors associated with such a dynamic environment.

## Tissue engineering

Tissue engineering is a branch of regenerative medicine and aims to create functional tissues through the combination of scaffolds, cells and other compounds. The aim is to repair or replace damaged native tissue.

The emergence of severe complications associated with PPL mesh has left surgeons with very limited options to treat POP, particularly when recurrent. There is now an unmet need for new treatments to fill the void left by PPL mesh and repair the weakened pelvic floor. Thus, there is a renewed focus on locating suitable replacements for PPL mesh to provide mechanical support but avoid the associated complications of pain and tissue erosion. This has stimulated researchers to look for alternative materials and there are a range of natural, synthetic, biodegradable or non-biodegradable options from which to choose.

The addition of cells to a material has been shown to improve the host response to that biomaterial in comparison with using the same material without cells added ^19^. Several different cell lines, including fibroblasts, smooth muscle cells and adult stem cells, have been proposed to use within the pelvic floor. Stem cells can be isolated from a variety of locations, including bone, blood, fat, skin, synovial fluid ^42^ and endometrium ^43, 44^. Mesenchymal stem cells (MSCs) are multipotent ^45^, meaning that they can differentiate into several tissue types, including fat, bone, cartilage, tendon and smooth muscle ^46^. MSCs have different paracrine effects which aid in wound healing ^47^. They can secrete immunomodulatory factors to encourage local tissue growth, control the inflammatory response, recruit neighbouring cells for tissue repair ^48^ and promote angiogenesis ^42^.

Ulrich *et al*. ^49^ demonstrated that endometrial MSCs seeded onto gelatin-coated polyamide mesh had an anti-inflammatory effect and promoted neovascularisation in a rat model. This study demonstrated that the meshes that had been seeded with cells also had significantly improved biomechanical properties with decreased stiffness and minimal fibrosis. Similarly, the addition of ADMSCs has been noted to improve the performance of different biomaterials in many other studies ^19, 21, 50^.

Within the pelvic floor, Wang *et al*. ^51^ have described a method of using pluripotent stem cell derivatives for the regeneration of the internal urethral sphincter. The pluripotent stem cells were induced into differentiation into a pure population of smooth muscle precursor cells. With a rat model, these precursor cells were injected peri-urethrally 3 weeks after inducing an acute sphincter injury. The study data demonstrated improvements in leak point pressures in the smooth muscle precursor cell–injected rats, consistent with restoration of urethral sphincter function. This work showed the potential for smooth muscle precursor cells, derived from pluripotent stem cells, to restore meaningful function to the internal urethral sphincter in acute injury. However, further studies have highlighted the limitations of injected cellular therapies for urinary sphincter deficiency with reduced efficacy seen when used to treat chronic pathology ^52^.

There is no question that cells can be implanted and proliferate in the presence of a suitable blood supply; the question is, will they go on to produce functional tissue at the target area of implantation? In the case of SUI, there may be denervation of the urinary sphincters and any resulting tissues derived from implanted cells are unlikely to become innervated. Equally, if the sphincters remain innervated, any subsequent tissues may not contract in a functional, physiological manner in synergy with bladder contraction to restore continence. Cell-based therapies have shown merit; however, at present, they are limited practically in their feasibility to translate to a workable product for use in an operating theatre. Using cells that have been manipulated in a laboratory and combining them with a material turn the material into an “advanced therapy medicinal product” ^53^, and much greater time and resources are required to produce them and there are greater regulatory hurdles to satisfy for market approval. With increasing research evidence demonstrating the value of cell-based therapies, perhaps the greater challenge is to overcome these practical limitations.

## Animal models

Increasingly, alternative methods of quantifying the biomechanics of the pelvic floor, including computational methods and animal models, have been used ^54^. Recent advances have helped us to better quantify the biomechanical properties of the female pelvic floor and in turn should lead to products with better *in vivo* predictability in the future. Lei *et al*. ^55^ characterised the biomechanical properties of vaginal tissue in women with POP both pre- and post-menopause. The authors found a significant difference in biomechanical properties between the POP and control groups in both pre- and post-menopausal women, suggesting that a degeneration of biomechanical properties of vaginal tissue is likely to be a precursor for the development of POP. Röhrnbauer *et al*. ^56^ described a new method for *in vivo* intravaginal measurement of the mechanical properties of the anterior vaginal wall. Using a novel aspiration device, they studied the degree of tissue displacement seen in women both with and without POP. The authors found that patients who had undergone an anterior colporrhaphy had statistically significantly reduced tissue displacement in comparison with pre-operative patients. A modified speculum to enable real-time measurement of vaginal biomechanics in an ovine model was described by Parkinson *et al*. ^57^. As these types of devices are trialled and developed, we may be able to reduce our reliance on animal models in the future.

Currently, animal models are an integral component of preclinical testing of new biomaterials. Several studies have looked at biomaterials implanted into the abdominal walls of rabbits. Roman *et al*. implanted PLA scaffolds into the abdominal walls of rabbits ^15^. PLA demonstrated better integration with host tissues in comparison with PVDF and PPL mesh. The commercial PVDF and PPL meshes both produced a chronic inflammatory response in the surrounding tissues. Conversely, the PLA and PU scaffolds showed evidence of constructive remodelling, showing an M2 macrophage response with angiogenesis ^15^. Clearly, the abdominal wall of a rabbit is not a perfect comparison for how biomaterials will behave within the female pelvic floor but this work has allowed the variety of implantable materials to be narrowed down to allow only those with a safe and stable profile to go forward for further testing.

Another animal model that would more closely mimic the anatomical, physiological and biomechanical properties of the human female pelvic floor was required. Sheep are known to develop POP after multiple births ^58^ and as such the sheep vagina has been identified as an appropriate model for evaluating the effects of different biomaterials ^31, 59^. Feola *et al*. ^31^ characterised the differences between the host response to PPL mesh when implanted in the vagina versus the abdominal wall in sheep. The authors found that vaginally placed mesh explants had double the contraction and greater stiffness and fibrosis in comparison with the same mesh implanted on the abdominal wall. Young *et al*. ^60^ studied the vaginal mechanical properties of both nulliparous and multiparous ewes through the use of a modified POP-quantification (POP-Q) score. Multiparous ewes were found to have patterns of vaginal wall weakness similar to those of women with increasing parity, suggesting that the former are a representative model for POP in humans.

## Mesh controversy

“Guidance for the preparation of a premarket notification application for a surgical mesh” was issued by the FDA in 1999 ^61^. This stated that any company proposing a new mesh device should include information regarding the tensile and burst strength (but incidentally gave no such requirement to detail cyclic or fatigue testing). However, at that time, little was known about the mechanical properties of the pelvic floor and what specification would constitute a “good” mesh product.

The initial uptake of PPL mesh amongst the urogynaecology community was extremely rapid, and a situation arose whereby there was an explosion of new devices on the market prior to full trial data becoming available. Indeed, trial data were sorely lacking as most devices were able to come to the market via the FDA 510(k) process, meaning that they had to prove only “substantial equivalence” with a previous product ^62^. In the case of pelvic mesh, this was the ProteGen sling, otherwise known as the grandfather mesh ^63^. This in turn was based on the Mersilene mesh (an interlocked polyester fibre) being used as its predicate, although this product was developed for hernia repair and had not been tested in the pelvic floor. This meant that there was no requirement to prove that these new products were safe for use in the pelvic floor. This system was changed in 2016, and all transvaginal mesh devices have been reclassified from class II (moderate-risk device) to class III (high-risk device), meaning that now the 510(k) process cannot be used for their introduction ^64^. Mesh manufacturers now have to provide detailed evidence confirming the safety and efficacy of their product, which was a key component that was lacking in the introduction of these products for use within the pelvic floor. The FDA state that they will continue to monitor the progress of patients with implanted transvaginal mesh through “post market surveillance measures” ^65^.

Further work has shown that the risk of mesh-related complications is highly dependent on the anatomical placement of the mesh in POP repair. We now know that abdominal repair is associated with much lower levels of complications (10% mesh exposure rate at 7 years ^66^) compared with transvaginal placement (12% exposure rate at 3 years ^67^ and 42% exposure rate at 7 years ^68^). Adverse events are lower still with mid-urethral slings for SUI, and tape-related complication rates are around 4% at 5 years ^69^. This includes mesh exposure rates of 4% ^70^ and rates of erosion into the viscera of less than 1% ^70^. It is likely that several interplaying factors, including a larger surface area of mesh being used, lead to increased erosion/exposure rates in POP. There is good evidence for a chronic inflammatory response to an implanted mesh, leading to tissue breakdown. However, it is not clear why this occurs in only some patients whereas others have a good clinical and functional outcome from the procedure. Certainly, we have learnt that the environment of the pelvic floor is vastly different from that of the abdominal wall and that different forces act upon it. Indeed, the vagina in particular is a highly mobile structure with the potential to undergo significant stress and strain through both sexual activity and childbirth. It is therefore imperative not to assume equivalent performance of the same mesh implanted in different sites of the body, which was the main assumption made with PPL mesh for abdominal hernia repair being placed into the pelvic floor ^71^.

In a 2017 joint consensus statement, the European Urology Association and the European Urogynaecological Association ^5^ reached the conclusion that synthetic mesh could be safely used in SUI surgery but that for prolapse repair its use should be reserved for complex cases in specialist centres. The Scottish government review ^72^ concluded that synthetic mesh procedures should still be offered for SUI but that they showed no additional benefit for POP. A recent update from the UK National Institute of Clinical Excellence (NICE) has recommended that transvaginal mesh repair of anterior and posterior vaginal wall prolapse be performed only in the context of research ^67^ but that synthetic mesh for SUI still be offered. All documents stressed the importance of informed consent with detailed explanations of risks to patients prior to the procedure.

## Discussion

There were several issues with the introduction of synthetic mesh for use within the pelvic floor. The FDA 510(k) route for introduction of products to market overestimated the safety of PPL mesh for pelvic floor repair because of their success in treating abdominal wall hernia repair. As such, there was no stipulation for long-term trials of pelvic floor mesh prior to their widespread adoption and abdominal meshes were deemed suitable predicates. Equally, at the time, little was known regarding the unique biomechanics of the pelvic floor and hence this was not given the adequate emphasis prior to approval of the pelvic floor mesh. If the behaviour of mesh had been predicted within the pelvic floor and new products had not been brought to market simply because of predicates, there may not have been such a rapid dissemination of products and widespread complications.

The introduction of any new materials for the pelvic floor clearly has to proceed with caution given the severity of the consequences of the use of vaginally inserted PPL mesh. Many women have been adversely affected by the placement of PPL mesh; as such, there is an understandable amount of scepticism towards the introduction of any new materials, particularly those of a synthetic nature. Any new materials need to demonstrate robust safety data in both preclinical and animal studies before proceeding to clinical trial and eventually to market.

It is clear that our knowledge of what constitutes an ideal biomaterial for use in the pelvic floor is still evolving, but new technologies are rapidly expanding our understanding of the biomechanical properties that they would have to withstand. A material should be strong enough to withstand dynamic distension yet not so rigid as to cause erosion through a patient’s native tissue. It should be biocompatible and not produce an intense inflammatory reaction leading to fibrosis. It must be a product that is acceptable to both patients and surgeons and that is feasible to insert in a single operation without requiring laboratory manipulation. Promising new avenues are being explored in tissue engineering with regard to new materials and cell therapies for use in the pelvic floor. New products that are likely to come to market will have to satisfy all regulatory hurdles and be acceptable to patients who are rightly sceptical given past product failings.

## Take-home messages

1. Because of aging populations, there is an increasing number of women with SUI or POP. This is combined with lifestyle (phenotypic) factors that contribute to its occurrence (age, body mass index, childbirth, smoking, among others) and genetic factors, such as increased breakdown of collagen or increased levels of proteolytic enzyme (for example, Ehlers-Danlos syndrome).

2. Thus, tissue-based repair has been challenging (bladder neck suspension for SUI and tissue plication for POP) with a high rate of recurrence.

3. The current PPL mesh use at the mid-urethra for SUI is the gold standard with long-term level-one evidence and mesh exposure of 3%, and erosion into the viscera of less than 1% ^70^. However, it is clear that complications were identified with PPL used for abdominal hernia repair and it was not evaluated biomechanically either in the laboratory or in animal models prior to being introduced into clinical practice for use within the pelvic floor.

4. Use of mesh in POP has improved anatomic success in level-one prospective trials; however, rates of complications and reoperations for mesh are more than 10% and this is related to the larger surface area of the mesh which is used ^70^.

5. Complications are much lower in surgeons/centres with female pelvic health training in high volume and this is the basis for a recent consensus statement ^73^.

6. Cell-based therapy for SUI has failed in clinical settings despite success in animal models. (Animal models sustain an acute injury whereas patients have a chronic injury.) Biomaterials and cell-based therapies conceptually offer an option but are still being developed (costs and regulatory approvals are currently prohibitive) and do not solve the patient-based tissue defect to replace this material. There is no commercially viable alternative to large-pore, light-weight PPL in patients with recurrent POP, but there is ongoing work to address this at present.

7. Synthetic material is widely accepted for SUI and abdominal (laparoscopic/robotic) use in women and hernia repair in men but is controversial via a vaginal approach for repair of POP. The European Urology Association, the FDA, the American Urology Association, the Society of Urodynamics, Female Pelvic Medicine and Urogenital Reconstruction (SUFU), and the American Board of Obstetrics and Gynecology policy statements suggest a dialogue with the patient regarding the evidence of risks involved and fully informed consent ^5^.

## Abbreviations

ADMSC, adipose-derived mesenchymal stem cell; FDA, US Food and Drug Administration; MSC, mesenchymal stem cell; PLA, polylactic acid; POP, pelvic organ prolapse; PPL, polypropylene; PU, polyurethane; PVDF, polyvinylidene fluoride; SUI, stress urinary incontinence
